# Heroin-Induced Leukoencephalopathy Leading to Locked-In Syndrome: A Case Report

**DOI:** 10.7759/cureus.38020

**Published:** 2023-04-23

**Authors:** Jurgen Shtembari, Dhan B Shrestha, Kaiyuan Zhang, Dinesh Rengarajan, Tilak Joshi

**Affiliations:** 1 Department of Internal Medicine, Mount Sinai Hospital, Chicago, USA; 2 Department of Medicine, Mount Sinai Hospital, Chicago, USA; 3 Department of Internal Medicine, Ross University School of Medicine, Barbados, BRB

**Keywords:** heroin intoxication, locked-in syndrome, chasing the dragon syndrome, heroin-induced leukoencephalopathy, heroin

## Abstract

Heroin-induced leukoencephalopathy (HLE) is a rare neurological sequela of heroin use. Heroin can be consumed through different routes such as inhalation, intravenous injection, and snorting. HLE cases have been reported via each route. However, heroin vapor inhalation has a higher rate of HLE and is also known as “chasing the dragon syndrome.”

We present a 65-year-old male who came unresponsive after heroin intoxication. During the hospital stay, he developed locked-in syndrome secondary to brain damage by HLE sequelae.

## Introduction

The first case of heroin-induced leukoencephalopathy (HLE) due to heroin inhalation was reported in the Netherlands in 1982 [1]. In a 2019 study of 50 cases of HLE, inhalation was found to be the leading route (60%), followed by intravenous injection (30%) and snorting (10%) [2]. Common clinical manifestations of HLE include focal neurological deficits, cerebellar ataxia, dysarthria, altered mental status, features of parkinsonism, and urinary or fecal incontinence [1,3,4]. The pathogenesis behind HLE remains uncertain. Brain biopsy has shown that mitochondrial changes and dysfunction may play a role in HLE [1].

The diagnosis of HLE is based on clinical and neuroimaging criteria. The proposed inclusive criteria include “signs and symptoms of HLE, positive heroin toxicology, witness or patient confirmation of heroin consumption, neuroimaging indication of HLE, and brain biopsy consistent with spongiform leukoencephalopathy." The exclusive criteria consist of “other confirmed toxins or substances known to produce similar clinical findings to HLE, evidence of infectious, demyelinating, vascular, or paraneoplastic etiology, and prominent cortical involvement in neuroimaging with relative sparing of the subcortical and posterior fossa areas” [5].

Diagnosing HLE can be challenging due to its clinical presentations and the lack of definitive biomarkers. Here, we present a case of HLE complicated by locked-in syndrome (LiS) and the diagnostic challenges we encountered.

## Case presentation

A 65-year-old male with a past medical history significant for chronic obstructive pulmonary disease, congestive heart failure, hypertension, and polysubstance use disorder presented to the emergency department with altered mental status. He became altered while working out after using heroin at home, as reported by the EMS. Further history taken from the family confirmed the EMS records, including witnessed use of heroin.

An initial neurological exam revealed a patient following simple commands with preserved motor strength in all extremities but verbally non-responsive. No significant head trauma was appreciated, and CT of the head showed no acute intracranial abnormalities such as hemorrhage, midline shift, or mass effect. However, his respiratory status deteriorated, and the patient was intubated for airway protection. The urine drug screen was positive for cocaine and benzodiazepines; however, there were no other remarkable findings upon initial work-up. A preliminary clinical suspicion was raised for acute metabolic encephalopathy due to substance use. The hospital course was complicated by sepsis and prolonged ICU stay requiring tracheostomy. While off sedatives, he remained significantly encephalopathic with a markedly changed neurological exam, positive only for the spontaneous opening of the eyes and positive corneal, cough, and gag reflexes.

**Figure 1:**
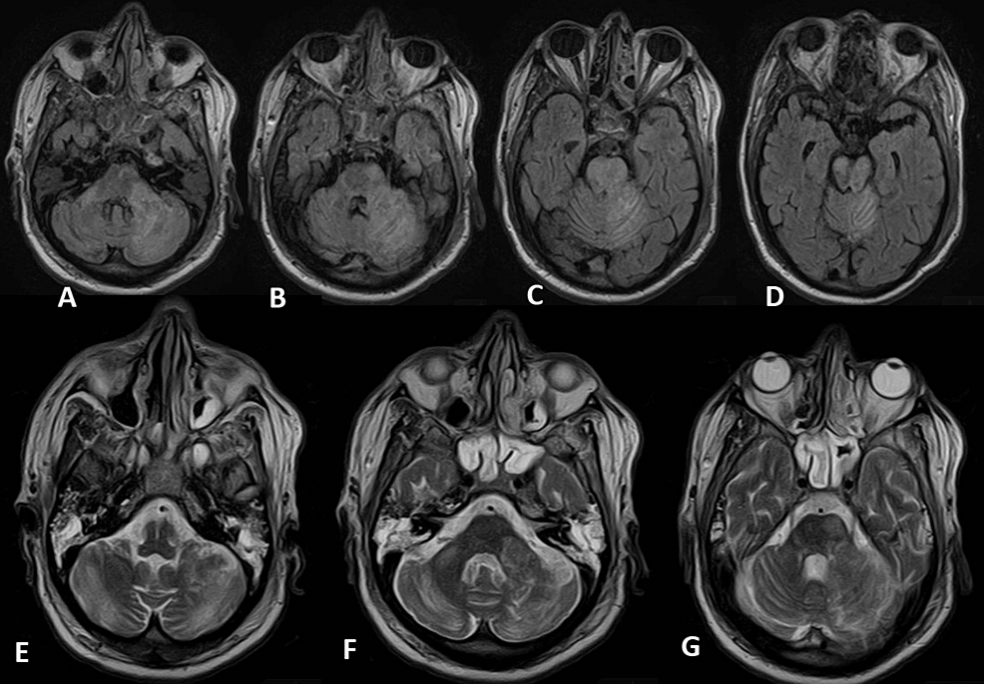
MRI images showing restricted diffusion scattered within bilateral pons and cerebellar peduncles (A-D) and a high signal within the bilateral pons and bilateral cerebellar peduncles bilateral cerebellar hemispheres on T2 (E-G)

MRI of the brain was obtained and showed normal arteries of the circle of Willis and extensive white brain matter changes. In addition, the MRI revealed fluid-attenuated inversion recovery (FLAIR) weighted sequences with restricted diffusion scattered within bilateral pons and cerebellar peduncles (Figure 1, A-D), a high signal within the bilateral pons and bilateral cerebellar peduncles, and bilateral cerebellar hemispheres on T2 (Figure 1, E-G). The MRI findings satisfied the imaging requirements for the diagnosis of HLE.

Given the extensive brainstem injury and his neurological exam with eye movements in the setting of delirium, LiS was suspected. After treatment of opioid withdrawal and delirium, our patient started to make meaningful eye contact by blinking to "yes" or "no" questions. A following neuropsychiatric exam and a normal awake EEG with an organized and developed 8Hz waveform, reactive to eye-opening and closure, confirmed the diagnosis.

Despite all medical efforts, we could not assess our patient’s level of understanding properly. Because of the significant brain damage and delirium, he remained unable to make decisions about goals of care. As a result, his family decided to transfer him to a long-term acute care facility.

## Discussion

In 2010, the Advisory Council on the Misuse of Drugs (United Kingdom) published a report considering the use of foil as an intervention to reduce the potential harm of intravenous heroin injection [6]. Using foil to produce heroin fumes for inhalation is considered a cleaner method of consumption, as it eliminates the risk of blood-borne viruses and germs associated with intravenous injection. However, a review has shown that heroin-induced HLE cases caused by heroin inhalation are on the rise [2]. Therefore, the neurological sequelae of heroin consumption by inhalation deserve greater attention.

The diagnostic MRI requirements for HLE include diffuse, symmetrical white matter hyperintensity on T2 and FLAIR sequences in the cerebellum, cerebral peduncles, posterior cerebrum, and posterior limbs of the internal capsule, with sparing of U-fibers [7,8]. The sparing of the anterior limb of the internal capsule differentiates it from other differential diagnoses such as toluene toxicity or reversible posterior leukoencephalopathy [8]. Therefore, the MRI findings of our patient are consistent with the diagnostic requirements.

The specific metabolite of heroin, 6-monoacetylmorphine, is detectable in urine two to eight hours after heroin consumption [5]. Additionally, the patient's family reported that he was found unconscious on site 20 minutes after he was last seen, and it was estimated that it took 30 minutes to transport him to the hospital. Based on this timeline, the time period from heroin consumption to urine toxicology sample collection was roughly two to three hours. Therefore, even though the patient had a negative opioid urine toxicology on admission, his family witnessed his heroin consumption. Our patient also had a clear history of heroin abuse, with multiple hospital admissions due to heroin intoxication in the past at our hospital.

HLE has rarely been associated with LiS sequelae in the past. Ischemic and hemorrhagic brain stroke was the most common etiology of LiS [9]. LiS could also be caused by brain trauma [9], brainstem infection [10], and toxins [11]. There have been reported cases of LiS due to cocaine or heroin consumption [11-13]. Despite his history of cocaine use, we attribute the neurological findings in our patient to heroin use, given the typical MRI imaging for HLE.

LiS has three main types. The classical form is defined by total immobility, with the preservation of the ability to perform vertical eye movements, blink, and maintain a normal level of consciousness. The incomplete form is defined as the classical form with small additional motor functions. The total immobility form is defined as complete body paralysis and loss of eye movement, with the preservation of cortical function examined via EEG [14]. The patient's ability to perform vertical eye movements despite quadriplegia, along with normal findings on an awake EEG indicating a normal level of consciousness, is consistent with the classical form of the condition.

Acute initial management of LiS should be focused on securing the patient’s airways, preserving hemodynamic stability, and removing or reversing the causative agent [14]. There have been no clearly defined treatments for HLE-induced LiS. Since HLE is the inciting event, treating HLE would be beneficial in aiding the reversal and recovery of LiS in such patients.

There has been no definite reported treatment for HLE. However, antioxidants have shown varying improvements. Coenzyme Q10 (300 mg four times per day) alone or coenzyme Q10 in combination with vitamin E (2000 mg daily) and vitamin C (2000 mg daily) could be potential options [3].

## Conclusions

Our case provides evidence to advocate for the consideration of LiS as a potential complication of toxic encephalopathy, highlighting the importance of early detection and management. It may also reinforce the need for early MRI imaging in patients with an undiagnosed cause of metabolic encephalopathy. There are no definitive treatments for HLE; however, antioxidants can be considered in addition to acute initial management.
